# Supplementary material for: Further discussion on the reaction behaviour of triallyl isocyanurate in the UV radiation cross-linking process of polyethylene: a theoretical study
Source: R Soc Open Sci. 2019 Sep 25;6(9):182196. doi: 10.1098/rsos.182196 (PMC6774971; doi:10.1098/rsos.182196)
Supplement: Supplementary Materials 1 [file rsos182196supp1.docx]

**Royal Society Open Science**

Electronic Supplementary Materials (ESM-1)

**Further Discussion on the Reaction Behavior of Triallyl Isocyanurate in the UV Radiation Cross-linking Process of Polyethylene: A Theoretical Study**

HUI ZHANG^1,*^, YAN SHANG^1^, HONG ZHAO^1,*^, XUAN WANG^1^, BAOZHONG HAN^1,2,*^, ZESHENG LI^3^

Corresponding author e-mail:

[hust_zhanghui11@hotmail.com](mailto:hust_zhanghui11@hotmail.com); [hongzhao@hrbust.edu.cn](mailto:hongzhao@hrbust.edu.cn); [hbzhlj@163.com](mailto:hbzhlj@163.com)

The optimized standard orientation of equilibrium geometries of the transition states at the B3LYP/6-311+G(d,p) level.

TSPBpH

6 -2.408233 0.961177 0.513286

6 -1.301838 0.316938 -0.062452

6 -1.481086 -0.939868 -0.660444

6 -2.737347 -1.540040 -0.673446

6 -3.823958 -0.902982 -0.075937

6 -3.655294 0.348714 0.519053

6 0.002559 1.031894 -0.083958

8 0.008271 2.273322 -0.126105

6 1.293540 0.295667 -0.022810

6 2.440823 0.918436 -0.539753

6 3.680017 0.295496 -0.454390

6 3.796485 -0.947790 0.169293

6 2.668247 -1.564271 0.708269

6 1.422357 -0.951118 0.608489

1 0.554347 -1.427144 1.046944

1 2.759221 -2.520162 1.211446

1 4.764779 -1.430393 0.241336

1 4.557285 0.779105 -0.868770

1 2.338749 1.889474 -1.008075

1 -2.269596 1.942036 0.951158

1 -4.499571 0.848299 0.980544

1 -4.799621 -1.375857 -0.079723

1 -2.868440 -2.503595 -1.152724

1 -0.645052 -1.434427 -1.139554

1 0.664101 3.112561 1.155061

TSPBpTAIC

7 2.139288 1.632402 0.680008

6 1.422346 0.626062 1.322515

7 1.802062 -0.691498 1.069518

6 2.882070 -1.031879 0.273211

7 3.518310 0.037951 -0.370586

6 3.240581 1.393283 -0.122885

8 0.188182 0.876665 1.744286

6 1.181287 -1.776414 1.868813

6 1.926784 -2.041102 3.148862

6 1.387666 -1.913527 4.358036

8 3.259355 -2.181857 0.141429

6 4.643496 -0.274851 -1.260737

6 4.273852 -0.433081 -2.712114

6 3.076801 -0.247563 -3.256555

8 3.927589 2.284047 -0.582438

6 1.789082 3.046558 0.957864

6 0.850818 3.623095 -0.068397

6 1.146199 4.682262 -0.816321

8 -1.587407 0.966181 -0.096310

6 -2.485606 0.118382 -0.312476

6 -3.902517 0.529361 -0.200986

6 -4.931421 -0.090583 -0.931874

6 -6.240532 0.371052 -0.838525

6 -6.547915 1.448623 -0.008127

6 -5.532923 2.076809 0.717186

6 -4.222184 1.631225 0.613117

6 -2.101054 -1.266600 -0.673102

6 -0.882462 -1.472921 -1.345113

6 -0.450901 -2.756762 -1.655383

6 -1.217573 -3.861227 -1.279596

6 -2.419152 -3.672270 -0.595312

6 -2.863205 -2.387710 -0.299063

1 1.333541 3.061641 1.948899

1 2.722089 3.607224 0.983580

1 -0.113581 3.132143 -0.169019

1 0.435987 5.085450 -1.529495

1 2.109332 5.177879 -0.746392

1 5.372226 0.531185 -1.156907

1 5.096311 -1.197760 -0.895893

1 5.111288 -0.722057 -3.344023

1 2.917985 -0.379616 -4.320065

1 2.217228 0.052199 -2.667134

1 0.154383 -1.482613 2.070958

1 1.180296 -2.660914 1.232871

1 2.957186 -2.368265 3.037952

1 1.953183 -2.133572 5.256601

1 0.360865 -1.587199 4.492602

1 -0.503297 0.882055 0.990600

1 -4.701134 -0.912630 -1.597713

1 -7.021234 -0.106783 -1.419533

1 -7.569952 1.802157 0.068009

1 -5.766822 2.917690 1.360375

1 -3.426690 2.122111 1.159578

1 -3.785272 -2.253647 0.252742

1 -3.007423 -4.528544 -0.285140

1 -0.876906 -4.863431 -1.513610

1 0.485440 -2.898586 -2.182706

1 -0.295602 -0.609706 -1.633674

TSPBpTMPTMA

6 4.738167 -2.467797 -0.924575

6 3.554134 -1.829354 -0.500175

6 2.398337 -2.621653 -0.333209

6 2.435644 -3.991825 -0.551219

6 3.619122 -4.611129 -0.960882

6 4.765883 -3.838342 -1.152800

6 3.460661 -0.378947 -0.297546

8 2.299341 0.151438 -0.419269

6 4.635098 0.441723 0.028022

6 5.696882 -0.060534 0.810488

6 6.776655 0.745182 1.147378

6 6.834844 2.068881 0.705651

6 5.790466 2.582008 -0.063981

6 4.698498 1.786685 -0.389374

8 1.626097 2.433950 -0.065810

6 0.382877 2.687316 0.131908

8 -0.420429 1.611583 0.191383

6 -1.765090 1.602801 -0.354124

6 -1.963988 0.290766 -1.138964

6 -1.765709 -0.905770 -0.197382

8 -2.854042 -0.936715 0.755705

6 -2.730258 -1.404973 2.025007

8 -3.653579 -1.257469 2.784369

6 -0.033974 4.045955 0.351742

6 -1.246656 4.381540 0.872028

6 0.999881 5.104624 0.037718

6 -3.390699 0.298146 -1.757246

8 -4.289174 1.152465 -1.027184

6 -5.378723 0.750706 -0.310732

8 -5.798154 1.489566 0.540994

6 -0.913465 0.202167 -2.281188

6 -1.063275 -0.966198 -3.266472

6 -6.076977 -0.536234 -0.648847

6 -6.343942 -0.855187 -1.918358

6 -6.589902 -1.306070 0.537301

6 -1.467330 -2.102537 2.457714

6 -1.062355 -3.217547 1.848068

6 -0.827440 -1.540207 3.698871

1 1.949194 1.360676 -0.196386

1 5.653837 -1.076359 1.182374

1 7.572399 0.343551 1.764906

1 7.680811 2.694724 0.966025

1 5.825949 3.608965 -0.410326

1 3.896138 2.200100 -0.985126

1 5.628691 -1.880632 -1.108312

1 5.683788 -4.305283 -1.492366

1 3.645200 -5.680641 -1.135648

1 1.539813 -4.585046 -0.401895

1 1.482668 -2.139451 -0.017053

1 -1.496716 5.422310 1.035554

1 -1.990487 3.656204 1.167385

1 1.879153 4.993214 0.677599

1 1.348305 5.030238 -0.995843

1 0.580626 6.099684 0.192604

1 -2.471869 1.663709 0.469844

1 -1.905570 2.468082 -1.002055

1 -0.966005 1.142741 -2.842240

1 0.082985 0.164020 -1.834942

1 -0.294927 -0.892075 -4.039855

1 -0.932840 -1.938089 -2.784571

1 -2.032516 -0.970599 -3.772905

1 -3.786249 -0.713896 -1.825315

1 -3.347279 0.722560 -2.762992

1 -6.950651 -1.721431 -2.159416

1 -6.004278 -0.250677 -2.751569

1 -7.289780 -0.688212 1.105952

1 -7.098090 -2.217208 0.216716

1 -5.775821 -1.562731 1.220118

1 -1.786008 -1.841074 -0.759478

1 -0.809070 -0.821513 0.315454

1 -0.220817 -3.785874 2.228796

1 -1.566294 -3.615421 0.974557

1 -0.470310 -0.519307 3.527984

1 -1.557247 -1.494661 4.511080

1 0.018618 -2.154317 4.010911

TSTAICH1

7 -0.418641 1.153444 0.338955

6 -0.952161 -0.093808 0.651522

7 -0.350640 -1.203419 0.081399

6 0.955762 -1.171687 -0.392440

7 1.565930 0.088310 -0.347272

6 0.892238 1.289799 -0.087745

8 -2.160173 -0.219004 1.075627

6 -1.131790 -2.469988 -0.058499

6 -2.587434 -2.249401 -0.417593

6 -3.566809 -3.079235 0.112403

8 1.523006 -2.158615 -0.813578

6 2.994450 0.177980 -0.733219

6 3.903532 0.204251 0.465633

6 4.828171 -0.719502 0.711638

8 1.430823 2.373228 -0.216170

6 -1.180324 2.377440 0.676981

6 -1.929015 2.923317 -0.508483

6 -3.251354 3.059193 -0.550267

1 3.206192 -0.682554 -1.363551

1 3.103984 1.093606 -1.314859

1 3.780562 1.050532 1.136723

1 5.478968 -0.652977 1.576230

1 4.966569 -1.574172 0.056823

1 -1.864097 2.121569 1.484680

1 -0.451728 3.105585 1.035256

1 -1.317033 3.235245 -1.350867

1 -3.749663 3.483078 -1.414690

1 -3.881701 2.753425 0.279020

1 -1.042795 -3.040013 0.870743

1 -0.611564 -3.025554 -0.837275

1 -2.761535 -1.822428 -1.405201

1 -4.572817 -3.080751 -0.287023

1 -3.387205 -3.668838 1.004527

1 -2.673522 -1.019124 0.431280

TSTAICH

6 0.971768 1.010883 0.102831

7 -0.307709 1.185719 -0.398565

6 -1.182580 0.138072 -0.684348

7 -0.690574 -1.139470 -0.406625

6 0.580100 -1.407108 0.097052

7 1.371592 -0.292673 0.358750

6 -0.776410 2.567729 -0.675366

6 -1.411426 3.215824 0.525208

6 -2.687586 3.586508 0.570922

8 -2.283605 0.322210 -1.155938

6 -1.584366 -2.296476 -0.660563

6 -2.316709 -2.735416 0.578512

6 -3.640500 -2.706956 0.700588

8 0.971680 -2.539534 0.296255

6 2.756011 -0.534652 0.840767

6 3.718352 -0.781059 -0.289249

6 4.773664 -0.011291 -0.538783

8 1.670618 1.988936 0.454026

1 -2.281562 -1.989425 -1.436506

1 -0.950475 -3.100580 -1.034861

1 -1.696906 -3.107146 1.390124

1 -4.132626 -3.053047 1.602510

1 -4.277760 -2.335948 -0.095891

1 3.049842 0.334571 1.423168

1 2.701152 -1.405234 1.493973

1 3.518331 -1.657082 -0.900508

1 5.458801 -0.236244 -1.348315

1 4.990664 0.868886 0.058345

1 -1.485330 2.496711 -1.497189

1 0.099692 3.131220 -0.997829

1 -0.755234 3.388094 1.375039

1 -3.103016 4.072748 1.446324

1 -3.360792 3.414401 -0.262836

1 1.540603 2.453804 1.749360

TSTMPTMA

6 4.542482 -1.369604 -0.673029

6 4.097683 -1.378367 0.766459

6 2.657663 -1.291150 1.071364

8 1.885483 -1.121684 -0.022978

6 0.456869 -1.040443 0.189284

6 -0.205198 -0.635581 -1.133707

6 -1.716817 -0.457802 -0.887366

8 -2.288752 -1.671017 -0.359579

6 -3.290394 -1.676333 0.563987

8 -3.625904 -2.727138 1.040953

6 4.949605 -1.434316 1.801460

8 2.178445 -1.284199 2.214622

6 0.333014 0.726591 -1.606177

8 0.132957 1.713742 -0.570462

6 0.544743 2.974132 -0.859668

6 0.344700 3.900655 0.300927

6 0.638665 5.189174 0.104472

6 0.094432 -1.635708 -2.286269

6 -0.124910 -3.128003 -2.007930

8 1.010824 3.277103 -1.932946

6 -3.955993 -0.367575 0.910538

6 -3.753131 0.168160 2.113614

6 -4.903458 0.196216 -0.117819

6 -0.156956 3.348216 1.608870

1 0.096834 -2.010678 0.531957

1 0.254891 -0.303303 0.965801

1 4.588914 -1.442996 2.821974

1 6.020625 -1.475778 1.641204

1 5.628921 -1.446750 -0.736056

1 4.103812 -2.203200 -1.228015

1 4.231027 -0.451480 -1.178324

1 -2.208666 -0.230658 -1.838497

1 -1.887127 0.365466 -0.193058

1 -4.255254 1.083193 2.409483

1 -3.093266 -0.291859 2.839978

1 -5.367505 1.112997 0.249914

1 -4.402631 0.421494 -1.064162

1 -5.695519 -0.526430 -0.339112

1 -0.513771 -1.337152 -3.148488

1 1.136634 -1.490808 -2.587384

1 0.105253 -3.701843 -2.909797

1 0.535424 -3.492418 -1.217168

1 -1.150997 -3.347840 -1.715227

1 -0.176757 1.055708 -2.515307

1 1.398905 0.659771 -1.823145

1 0.522577 5.918829 0.897650

1 1.003280 5.532858 -0.855646

1 -0.243254 4.145513 2.348948

1 -1.136082 2.875667 1.493505

1 0.519758 2.584751 2.002881

1 2.321561 -0.027088 3.035147

TS1

6 2.920794 2.655567 -2.258800

6 1.628935 2.811626 -1.501473

6 0.470483 3.139956 -2.077903

6 1.760031 2.748428 -0.003469

8 2.679719 3.281209 0.564314

8 0.812114 2.122211 0.740745

6 -0.089699 1.119179 0.224053

6 -0.304000 0.062529 1.321610

6 -0.803496 0.689777 2.652913

6 -2.041458 1.591315 2.590706

6 -1.244691 -1.028494 0.780376

8 -2.449499 -0.443995 0.245352

6 -3.298456 -1.279430 -0.422660

6 -4.511913 -0.608577 -0.928799

6 -5.014464 0.591146 -0.167361

6 1.031736 -0.623487 1.662593

8 1.593616 -1.214589 0.468252

6 2.689450 -1.996143 0.642910

8 3.179763 -2.202815 1.727412

8 -2.777976 -2.378055 -1.055723

6 3.184056 -2.549779 -0.659506

6 2.421453 -2.256333 -1.924462

6 4.296100 -3.289032 -0.620567

6 -5.126159 -1.094495 -2.019559

1 -1.036833 1.589535 -0.034819

1 0.327458 0.658092 -0.670662

1 -0.438593 3.290267 -1.508735

1 0.406477 3.301367 -3.148548

1 2.761386 2.787065 -3.330117

1 3.652248 3.388995 -1.911579

1 3.359643 1.666872 -2.090005

1 -1.519081 -1.717053 1.590223

1 -0.753677 -1.600223 -0.004944

1 -6.030267 -0.633219 -2.399174

1 -4.734871 -1.955137 -2.546472

1 -5.946338 0.954533 -0.602921

1 -5.199092 0.346579 0.882873

1 -4.283397 1.403456 -0.181458

1 -0.993884 -0.132180 3.353188

1 0.022056 1.268988 3.075551

1 -2.275759 1.956807 3.594089

1 -1.870007 2.468648 1.962116

1 -2.916490 1.065327 2.208714

1 0.891959 -1.406001 2.411870

1 1.739905 0.104619 2.059177

1 4.714686 -3.722546 -1.521655

1 4.805199 -3.471344 0.317743

1 2.907185 -2.729815 -2.779296

1 1.393662 -2.624589 -1.864475

1 2.361415 -1.181082 -2.113049

1 -3.459081 -2.492223 -0.014062

TS1-1

6 2.772511 2.612842 -2.036082

6 1.408740 2.701555 -1.404173

6 0.280217 2.863801 -2.097913

6 1.415521 2.762164 0.099192

8 2.244833 3.406529 0.691789

8 0.455608 2.125423 0.817422

6 -0.323675 1.008021 0.334090

6 -0.366089 -0.063964 1.436602

6 -0.877155 0.502679 2.789046

6 -2.248584 1.187459 2.795212

6 -1.202429 -1.265699 0.943351

8 -2.528114 -0.902331 0.507796

6 -2.818435 -1.091104 -0.836807

6 -4.271785 -0.767355 -1.120462

6 -5.212079 -0.551477 0.038307

6 1.052831 -0.590262 1.716994

8 1.631571 -1.097555 0.492747

6 2.791208 -1.790202 0.611469

8 3.331448 -1.988616 1.674100

8 -1.938218 -1.178554 -1.705530

6 3.285457 -2.264067 -0.722741

6 2.475330 -1.968876 -1.957336

6 4.440791 -2.935013 -0.737522

6 -4.653122 -0.638647 -2.425281

1 -1.329846 1.366596 0.116399

1 0.104534 0.599583 -0.578381

1 -0.686306 2.959488 -1.618540

1 0.295152 2.929596 -3.180340

1 2.698929 2.642192 -3.124347

1 3.401157 3.438841 -1.695659

1 3.281113 1.688100 -1.745319

1 -1.335229 -1.970558 1.769319

1 -0.689258 -1.760995 0.120440

1 -5.681005 -0.408231 -2.675354

1 -3.936617 -0.767854 -3.225724

1 -6.244391 -0.522847 -0.313763

1 -5.122146 -1.333946 0.792531

1 -4.983915 0.396071 0.533514

1 -0.888661 -0.324149 3.509391

1 -0.133013 1.216973 3.151587

1 -2.475563 1.537459 3.805925

1 -2.267038 2.063225 2.141453

1 -3.046923 0.513258 2.482963

1 1.035676 -1.392083 2.458706

1 1.688099 0.210217 2.099231

1 4.861290 -3.308772 -1.664166

1 4.984193 -3.119813 0.180901

1 2.959768 -2.392254 -2.838923

1 1.465750 -2.381113 -1.883826

1 2.359698 -0.892556 -2.110001

1 -3.587968 -2.176735 -0.890298

TS2

6 3.264391 2.397405 -2.195612

6 1.979309 2.691283 -1.468177

6 0.900031 3.215562 -2.053548

6 2.048325 2.521432 0.025463

8 3.018219 2.870209 0.648726

8 0.990002 2.008588 0.709353

6 -0.034166 1.185220 0.117174

6 -0.413995 0.050557 1.092806

6 0.829950 -0.742110 1.516496

8 1.451568 -1.308914 0.338215

6 2.527554 -2.108282 0.561679

8 2.945077 -2.344088 1.669762

6 -1.158057 0.557268 2.361735

6 -0.367523 1.387753 3.382004

6 -1.343103 -0.913845 0.336086

8 -2.531611 -0.183074 -0.092085

6 -3.565227 -0.880927 -0.514693

6 -4.887375 -0.339112 -0.709009

6 -5.380931 0.763447 0.208291

8 -3.524030 -2.158840 -0.672557

6 3.096833 -2.639062 -0.718979

6 2.401356 -2.335024 -2.019732

6 4.211296 -3.370190 -0.629683

6 -5.509877 -0.485513 -2.004377

1 -0.909865 1.802634 -0.087754

1 0.325048 0.764440 -0.821894

1 0.001052 3.459632 -1.500816

1 0.897412 3.447589 -3.113057

1 3.167203 2.616191 -3.260063

1 4.078130 2.995172 -1.778774

1 3.552122 1.347585 -2.079375

1 -1.658800 -1.741494 0.974397

1 -0.862370 -1.322323 -0.551563

1 -6.561199 -0.244355 -2.099095

1 -5.060106 -1.092320 -2.779695

1 -5.049835 1.748784 -0.140155

1 -6.473361 0.772491 0.218574

1 -5.035972 0.631560 1.236348

1 -2.024356 1.132658 2.023815

1 -1.564946 -0.324542 2.872147

1 -1.029675 1.668338 4.205717

1 0.465209 0.828170 3.814719

1 0.039003 2.299268 2.945446

1 0.564718 -1.551710 2.200953

1 1.551866 -0.096818 2.014116

1 4.682415 -3.787080 -1.512464

1 4.669816 -3.562414 0.332398

1 2.941049 -2.786009 -2.854035

1 1.378811 -2.723001 -2.022663

1 2.332567 -1.258043 -2.194093

1 -4.802479 -1.830619 -0.581996

TS3

6 2.912393 2.666611 -2.198189

6 1.664909 2.848513 -1.375386

6 1.850287 2.682337 0.109647

8 0.908840 2.038557 0.845798

6 -0.038714 1.099100 0.291142

6 -0.274268 -0.012038 1.328297

6 -0.745018 0.554419 2.696914

6 -1.987742 1.452036 2.702312

6 0.506355 3.283150 -1.876231

8 2.807812 3.148323 0.673248

6 -1.252280 -1.045266 0.742236

8 -2.434963 -0.390548 0.234433

6 -3.331507 -1.206794 -0.392790

8 -3.158128 -2.405125 -0.517719

6 1.044102 -0.752517 1.618481

8 1.586260 -1.280394 0.385540

6 2.696120 -2.052400 0.502820

6 3.169275 -2.544750 -0.831962

6 4.299218 -3.257084 -0.850067

6 -4.515056 -0.479173 -0.865811

6 -5.461732 -1.234619 -1.722128

6 -4.600293 1.025583 -0.833361

8 3.214344 -2.296364 1.566258

6 2.366215 -2.224684 -2.065034

1 -0.971989 1.616678 0.076572

1 0.348140 0.675962 -0.635490

1 -0.366580 3.449092 -1.256657

1 0.408139 3.519799 -2.930224

1 2.720708 2.887698 -3.249296

1 3.704278 3.323743 -1.831246

1 3.290031 1.641843 -2.120402

1 -1.545026 -1.766419 1.510561

1 -0.786925 -1.598632 -0.073849

1 -6.273486 -0.703745 -2.196829

1 -5.265970 -2.273984 -1.937468

1 -5.639834 1.354447 -0.911637

1 -4.175147 1.437889 0.080593

1 -4.058860 1.454423 -1.685218

1 -0.917961 -0.297978 3.364155

1 0.088211 1.117686 3.125968

1 -2.192842 1.783270 3.723812

1 -1.840521 2.350226 2.097312

1 -2.872663 0.933372 2.331680

1 0.883567 -1.579114 2.314515

1 1.773724 -0.072841 2.059168

1 4.703644 -3.646231 -1.777504

1 4.837336 -3.461780 0.067188

1 2.839905 -2.654732 -2.949122

1 1.349972 -2.621475 -1.991048

1 2.274796 -1.145179 -2.212471

1 -5.521549 -1.054164 -0.378865

TSTAC1

7 -0.204902 1.325825 -0.262620

6 1.042339 1.166260 0.289824

7 1.561538 -0.150877 0.314455

6 0.807016 -1.272747 0.015010

7 -0.513445 -1.016554 -0.364318

6 -0.981125 0.286965 -0.548049

8 1.693046 2.098172 0.725972

6 2.962381 -0.358093 0.745608

6 3.906844 -0.477645 -0.420026

6 4.904616 0.371157 -0.649984

8 1.247266 -2.405393 0.089191

6 -1.383808 -2.174590 -0.660826

6 -2.085831 -2.689407 0.566670

6 -3.407461 -2.786783 0.681435

8 -2.164331 0.421243 -0.996295

6 -1.405837 3.028321 0.650917

6 -2.506151 2.982106 -0.168677

6 -3.273661 1.818046 -0.139771

1 3.222823 0.493650 1.370012

1 2.978828 -1.271254 1.342256

1 3.742145 -1.325168 -1.080252

1 5.576781 0.241109 -1.490812

1 5.081148 1.227074 -0.006111

1 -2.100610 -1.858839 -1.415601

1 -0.736029 -2.948680 -1.074328

1 -1.439765 -3.016490 1.377655

1 -3.870854 -3.194142 1.572997

1 -4.071923 -2.471303 -0.117329

1 -1.369817 2.447093 1.563716

1 -0.625703 3.768987 0.536129

1 -2.559939 3.630191 -1.035665

1 -4.074207 1.660125 -0.849727

1 -3.339008 1.235170 0.770719

TSTAC2

7 -0.227636 0.736747 -0.474173

6 -1.040441 -0.314086 -0.203690

7 -0.707329 -1.497219 0.222459

6 0.618170 -1.730367 0.513073

7 1.502331 -0.621852 0.367556

6 1.075321 0.577539 -0.168159

8 -2.328502 -0.027236 -0.442062

8 1.053267 -2.799379 0.891486

8 1.917335 1.509310 -0.340475

6 -0.942126 2.759134 0.107164

6 0.141080 3.182928 0.846713

6 1.336496 3.356787 0.153667

1 -0.997770 2.967108 -0.951906

1 -1.861347 2.421927 0.569339

1 0.156589 3.043772 1.922016

1 2.262576 3.563114 0.673791

1 1.315821 3.623387 -0.894363

6 -3.300239 -1.084467 -0.223983

6 -4.637429 -0.550203 -0.624668

1 -3.009459 -1.945278 -0.832018

1 -3.275760 -1.391001 0.823279

6 -5.706133 -0.562784 0.166897

1 -4.710518 -0.165665 -1.638953

1 -6.670601 -0.206390 -0.176199

1 -5.656905 -0.940175 1.183808

6 2.930538 -0.829707 0.684394

1 3.358032 0.140825 0.927641

1 2.955875 -1.473851 1.564553

6 3.683629 -1.477320 -0.447109

6 4.730746 -0.924674 -1.052530

1 3.331392 -2.462042 -0.740869

1 5.260433 -1.437400 -1.847678

1 5.095611 0.059696 -0.775532

TSTAC

7 -1.007187 1.002166 -0.492840

6 -0.939088 -0.328282 -0.342942

7 0.159317 -1.047612 -0.247248

6 1.284288 -0.299616 -0.219928

7 1.365033 1.010612 -0.276950

6 0.190040 1.676058 -0.463565

8 -2.134955 -0.929949 -0.305983

8 2.396063 -1.030468 -0.106669

8 0.208121 2.934770 -0.611110

6 -2.594916 2.060915 0.425389

6 -1.891734 3.052103 1.090919

6 -1.228703 3.973559 0.287909

1 -3.016485 2.261065 -0.550203

1 -3.012200 1.207135 0.944234

1 -1.557346 2.887243 2.109371

1 -0.532045 4.686633 0.708749

1 -1.637109 4.233504 -0.679535

6 -2.158451 -2.376132 -0.170234

6 -3.587770 -2.807124 -0.243325

1 -1.566453 -2.805584 -0.982858

1 -1.691459 -2.661324 0.774257

6 -4.181885 -3.556680 0.680688

1 -4.132466 -2.495375 -1.130987

1 -5.209013 -3.884822 0.570447

1 -3.659174 -3.876387 1.577045

6 3.662525 -0.326145 -0.081661

6 4.746184 -1.355316 -0.048848

1 3.727172 0.297454 -0.978132

1 3.697677 0.333567 0.787596

6 5.732263 -1.363082 0.843303

1 4.708210 -2.107404 -0.832806

1 6.524613 -2.101788 0.806721

1 5.785583 -0.626461 1.639243
