# Supplementary material for: Further discussion on the reaction behaviour of triallyl isocyanurate in the UV radiation cross-linking process of polyethylene: a theoretical study
Source: R Soc Open Sci. 2019 Sep 25;6(9):182196. doi: 10.1098/rsos.182196 (PMC6774971; doi:10.1098/rsos.182196)
Supplement: Supplementary Materials 2 [file rsos182196supp2.docx]

**Royal Society Open Science**

**Electronic Supplementary Materials (ESM-2)**

The schematic diagrams of reaction progress of the studied thirteen channels.
